# Outlines of the Science and Practice of Medicine

**Published:** 1875-03

**Authors:** 


*Outlines of the Science and Practice of Medicine.* By William Aitken, M. D., F. R. S. London: Charles Griffin & Co. Philadelphia: J. B. Lippincott & Co., 1874. Buffalo: Martin Taylor.

Dr. Aitken is so well known through the medium of his work—The Science and Practice of Medicine—that anything from his pen will naturally be expected to be of a high order. In this work he has not attempted to give to the student a hand-book to prepare him for examinations, etc., but has, on the contrary, placed before him the outlines of the various subjects which he will be expected to pursue in order to obtain a knowledge of the Science and Practice of Medicine.

The work is divided into three parts which consider: I. Topics relative to Pathology; II. Methodical nosology, systematic medicine or the distinctions and definitions, the nomenclature and classification of diseases; III. The nature of diseases, special pathology and therapeutics.

Chapter II. of Part I. is especially valuable, and should commend itself to the attention of all students as well as to many practitioners; it relates to the methodical examination of patients and recording medical cases. Students are expe ted to know something about this important subject, and we are pleased to see it introduced into a book intended especially for them.

The methodical arrangement of the book is admirable, and it seems in every particular to carry out the import of its name—the "Outlines of the Science and Practice of Medicine." It contains all that the student needs in a work of its character, omitting all that he is expected to learn from more pretentious treatises.

